# Autologous chondrocyte implantation (ACI) for the treatment of large and complex cartilage lesions of the knee

**DOI:** 10.1186/1758-2555-3-11

**Published:** 2011-05-21

**Authors:** Christian Ossendorf, Matthias R Steinwachs, Peter C Kreuz, Georg Osterhoff, Andreas Lahm, Pascal P Ducommun, Christoph Erggelet

**Affiliations:** 1 Department of Surgery, Division of Trauma Surgery, University Hospital Zurich, Raemistrasse 100, 8091 Zurich, Switzerland; 2 Division of Orthobiology and Cartilage Repair, Schulthess Clinic, Lengghalde 2, 8008 Zurich, Switzerland; 3 Department of Orthopaedics, University of Rostock, Doberaner Strasse 142, 18057 Rostock, Germany; 4 Department of Orthopaedics, University of Greifswald, F.-v.-Sauerbruchstr., 17475 Greifswald, Germany; 5 Department of Surgery, Division of Plastic, Aesthetic and Hand Surgery, University Hospital Zurich, Raemistrasse 100, 8091 Zurich, Switzerland; 6 Department of Orthopaedics and Trauma Surgery, University of Freiburg, Hugstetter Str. 55, 79106 Freiburg, Germany

## Abstract

**Background:**

Complex cartilage lesions of the knee including large cartilage defects, kissing lesions, and osteoarthritis (OA) represent a common problem in orthopaedic surgery and a challenging task for the orthopaedic surgeon. As there is only limited data, we performed a prospective clinical study to investigate the benefit of autologous chondrocyte implantation (ACI) for this demanding patient population.

**Methods:**

Fifty-one patients displaying at least one of the criteria were included in the present retrospective study: (1.) defect size larger than 10 cm^2^; (2.) multiple lesions; (3.) kissing lesions, cartilage lesions Outerbridge grade III-IV, and/or (4.) mild/moderate osteoarthritis (OA). For outcome measurements, the International Cartilage Society's International Knee Documentation Committee's (IKDC) questionnaire, as well as the Cincinnati, Tegner, Lysholm and Noyes scores were used. Radiographic evaluation for OA was done using the Kellgren score.

**Results and Discussion:**

Patient's age was 36 years (13-61), defects size 7.25 (3-17.5) cm^2^, previous surgical procedures 1.94 (0-8), and follow-up 30 (12-63) months. Instruments for outcome measurement indicated significant improvement in activity, working ability, and sports. Mean ICRS grade improved from 3.8 preoperatively to grade 3 postoperatively, Tegner grade 1.4 enhanced to grade 3.39. The Cincinnati score enhanced from 25.65 to 66.33, the Lysholm score from 33.26 to 64.68, the Larson score from 43.59 to 79.31, and Noyes score from 12.5 to 46.67, representing an improvement from Cincinnati grade 3.65 to grade 2.1. Lysholm grade 4 improved to grade 3.33, and Larson grade 3.96 to 2.78 (Table 1), (p < 0.001). Patients with kissing cartilage lesions had similar results as patients with single cartilage lesions.

**Conclusion:**

Our results suggest that ACI provides mid-term results in patients with complex cartilage lesions of the knee. If long term results will confirm our findings, ACI may be a considered as a valuable tool for the treatment of complex cartilage lesions of the knee.

**Table 1:** Mean scores and grades at surgery (Tx) and at follow-up

	Tx		Follow-up	
	Score	Grade	Score	Grade
ICRS		4		3
Tegner		1		3
Noyes	13		47	
Cincinnati	26	4	66	2
Lysholm	33	4	65	3
Larson	44	4	79	3

## Introduction

Autologous chondrocyte implantation (ACI) has been recommended for the treatment of symptomatic cartilage defects of approximately 2.5-10 cm^2^. Smaller defects are usually treated by microfracture resulting in fibrocartilaginous repair tissue with only limited durability [1-4], while ACI provides hyaline like cartilage [4]. Large cartilage defects, kissing lesions and OA cannot be addressed by microfracture. In addition, cartilage defects are frequently accompanied by anterior cruciate ligament (ACL) tears, meniscus injuries, or malalignment. Hence, these defects represent a challenging task for the orthopaedic surgeon, as -particularly in younger patients- surgical treatment options providing proper knee joint function at follow-up are usually limited. Arthroscopic debridement was shown to be insufficient for the treatment of mild and moderate OA. Total knee arthroplasty (THA) provides good results in elderly. However, in younger patients, the outcome of THA is less favorable. Moreover, due to the young age of patients, revision surgery will be unavoidable after several years. Patients with the profile described above, usually physically active, frequently present with sometimes yearlong and often immobilizing pain. Often, they have a history of cartilage debridement and microfracture and/or various knee surgeries before presentation at our department. Sporadically, we performed ACI in patients with complex and large cartilage lesions of the knee. Follow-up examinations revealed encouraging results. Therefore, we were prompted to assess ACI in this challenging group of patients.

## Materials and methods

**Figure 1:**
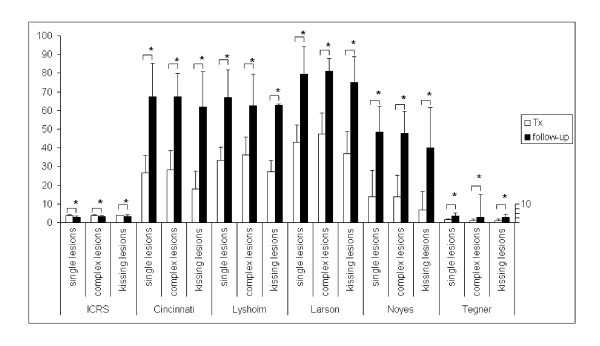
**Patients were divided into 3 groups: single lesions (n = 23), complex lesions (n = 19), and kissing lesions (n = 9) at surgery (Tx) and at follow-up**. Values are reported as mean+/-standard deviation with * indicating statistically relevant changes.

Fifty-one patients (18 female, 33 male; age 36 (13-61) years; 78 cartilage defects) with at least one of the following criteria were included in the present retrospective study: cartilage defect larger 10 cm^2^, multiple lesions, kissing lesions, and OA, respectively. All but 5 defects were classified as Outerbridge [5] Grade IV, mainly on the medial femoral condyle (n = 35), whereas 10 lesions were on the lateral femoral condyle, and 15 on patella and trochlea, respectively (Figure 1, Table 2). Mean defects size was 7.25 (3-17.5) cm^2^. Average follow-up was 30 months (12-63). In 33 cases, the defects were situated on the right knee, in 18 cases on the left. Fourteen patients showed traumatic defects; 22 degenerative defects, and 15 patients had osteochondritis dissecans (OD) lesions. Number of previous surgeries was 1.94 (0-8). In addition to ACI, concomitant surgical procedures as anterior cruciate ligament (ACL) reconstruction (n = 1), patella realignment surgery (n = 1), high tibial osteotomy (HTO), (n = 8), microfracture (n = 3), and osteochondral autograft transfer (OATS), (n = 11) were done in 23 patients (Table 2). Patients were divided into 3 groups: single lesions (n = 23), complex lesions (n = 19), and kissing lesions (n = 9).

**Table 2:** Patient's characteristics

Characteristic	
Gender	33 male, 18 female
Age (years)	36 (range 13-61)
Height (cm)	176 (range 140-196)
Weight (kg)	74 (range 45-98)
Treated knee	33 right, 18 left
Defect size (cm^2^)	7.2 (3-17.5)
Cartilage grade	1 grade III, 50 grade IV (1st lesion); 4 grade III, 23 grade IV (2nd lesion)
Localization (1st lesion)	32 medial, 7 lateral, 5 patella, 7 intercondylar notch
Etiology	14 traumatic, 22 degenerative, 15 OD
Number of previous surgical procedures	7 × 0, 18 × 1, 11 × 3, 4 × 4, 1 × 5, 1 × 7, 1 × 8
Additional procedures	1 ACL-reconstructions, 1 Ali Crogius, 8 HTO, 3 microfractures, 11 OATS
Previous surgical procedures	20 meniscectomies, 5 ACL-reconstructions, 5 lateral releases, 14 drilling/microfracture, 7 abrasion arthroplasty

Preoperative leg axis was determined in every case. Malalignment was corrected in the same session with ACI. In 1 patient with ACL-lesion, ACL-repair was done prior to ACI in the same operation using a graft. One patient had an Ali Crogius procedure due to femoro-patellar instability. None of the patients had concomitant meniscus surgery. No patient with severe varus/valgus deformity was excluded from the present study. For evaluation of treatment outcome, ICRS [6], Cincinnati [7-9], Tegner [9], Lysholm [10], Larson [11] and the Noyes [7] scores were used. For radiographic evaluation, the Kellgren radiographic score was applied [12,13]. The present study was approved by the local ethical commission. All patients gave their informed consent to participate in the study. All patients were examinated by an investigator independent from the surgical and outpatient teams, respectively. Statistical analysis was performed using the paired Wilcoxon-rank sum test with SPSS for Windows 11.5 (SPSS, Chicago, U.S.A.). The level of significance was set to 5%.

### Surgical technique

For ACI, the cartilage defect was assessed arthroscopically for definite indication to ACI. Approximately 250 mg of articular cartilage was taken as a biopsy from a lesser or non weight bearing region of the knee as the linea terminalis or the intercondylar notch. The biopsy was placed in transport container provided by the commercial cell culturing company and sent to the company's cell culturing facility (Genzyme Biosurgery, Cambridge, MA, U.S.A.). There, chondrocytes were expanded *in vitro *and brought into a suspension for later injection. Approximately three to six weeks later the implantation of the cultured autologous chondrocytes was performed. Under general anesthesia and antibiotic prophylaxis, a standard arthrotomy was performed preparing the cartilage defect in a tourniquet-controlled bloodless field. The cartilage lesion was carefully debrided back to healthy cartilage building a stable rim. A template was fitted to defect size and periosteum was harvested from the lateral aspect of the tibia using this template. This periosteal flap was fitted to defect size and sutured into the defect cambium layer down (Vicryl 6-0), leaving a gap for the injection of the cultured chondrocytes. The rim was sealed with fibrin glue. The chondrocyte suspension was injected under the periosteal flap and the flap was finally secured and sealed with fibrin glue and a final suture.

## Results

No patient was lost to follow-up. Postoperatively, no knee joint infection occurred. Two patients showed an extension deficit of 5°. No flexion deficiency could be observed at latest follow-up. All patients were able to bend and flex the knee operated on to at least 120° Overall, mean ICRS grade improved from 3.8 preoperatively to grade 3 postoperatively, Tegner grade 1.4 enhanced to grade 3.39. The Cincinnati score enhanced from 25.65 to 66.33, the Lysholm score from 33.26 to 64.68, the Larson score from 43.59 to 79.31, and Noyes score from 12.5 to 46.67, representing an improvement from Cincinnati grade 3.65 to grade 2.1. Lysholm grade 4 improved to grade 3.33, and Larson grade 3.96 to 2.78 (Table 1). All score improvements were statistically relevant (p < 0.001).

Average grade of OA was 1.28. No signs of OA could be determined on plain radiographs in 17 patients, initial OA was present in 5, mild in 25 and moderate in 3 patients.

**Figure 2:**
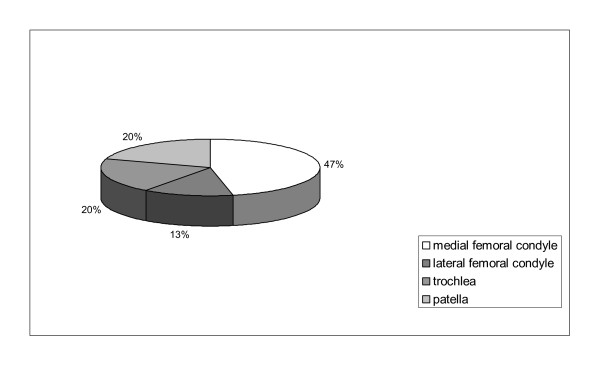
**Cartilage defects were situated mainly on the medial femoral condyle (n = 35), whereas 10 lesions were on the lateral femoral condyle, and 15 on the patella and on the trochlea, respectively**.

Results in patients with complex defects and such displaying kissing lesions were not worse than patients with single lesions (Figure 2). Evaluated by using the ICRS and Tegner score, patients of all 3 groups showed a high level of resemblance (Figure 2). Noyes and Cincinnati scores showed nearly similar patterns of improvement for single, complex and kissing lesions. Within the respective groups, scoring results of single lesions resembled those of complex lesions, both, preoperatively and at follow-up. Patients with kissing lesions scored better than those of the other groups when using the Lysholm and Larson score, while patients with complex lesions scored worse (Figure 2), although the differences were not statistically relevant.

During the follow-up period, 27 patients required reoperations mostly due to persistent symptoms like locking or catching sensations, pain or swelling. The outcome of these reoperations comprised shavings/debridements (n = 23), synovectomies (n = 5), microfracture (n = 4), meniscectomies (n = 3), autologous chondrocyte implantation as revision procedure (n = 3), and patella realignment surgery (n = 1), (Table 3). Reoperations were recorded regardless whether the adverse event was related to ACI. Two patients required total knee arthroplasty (THA) later on.

**Table 3:** Reoperations in 27 patients

Procedure	
Shaving	23
Synovectomy	5
Microfracture	4
Meniscectomy	3
ACI	3
Ali Crogius	1

## Discussion

The aim of the present study was to evaluate ACI in patients with complex and large cartilage lesions of the knee such as large defects, multiple lesions, kissing lesions or OA. Highly significant improvements in activity level and in sports were observed. A relatively high number of patients required revision surgery due to locking or catching sensations. Potential weaknesses of the present study emerge from the context of heterogeneous patient population including patient's age and number of previous surgical procedures. No biopsies were available for analysis of replacement tissue quality.

Evolution of ACI technology with matrix-induced ACI (MACI) potentially comprises less morbidity, as no periosteum has to be harvested [14]. In contrast, periosteum alone was shown to be capable to promote cartilage formation [15,16]. Therefore, the proportion of either the periosteal graft or the chondrocyte suspension to formation of the repair tissue remains to be clarified. Some authors recommend ACI as a salvage procedure or treatment option in the arthritic knee [17]. Here, significant improvements in quality of life, pain relief, and in activity in patients with multiple lesions alone were shown. Patients with high tibial valgus osteotomy and such with patellofemoral defects were doing worse. In the present study we could not confirm these findings, as no significant differences in our patients with the named conditions could be observed. This might be caused by the smaller number of patients in our study and the consecutive statistic effects. Other authors considered ACI as unsuitable for OA [2]. In contrast, recent studies successfully used ACI in the treatment of early stage and/or early OA [4,18].

In the evaluation of cartilage repair procedures there are basically two methods: biopsy and the use of scoring systems, each of which has specific advantages and disadvantages. Though a biopsy provides a clear picture of the type and quality of repair tissue, it is difficult to gather specimens from asymptomatic and well-being patients.

One score alone does not cover all the desired facts, so that at least two or more scores have to be used. We may speculate that our encouraging mid-term results will represent decent long-term outcome also in the future. Nevertheless, status at two years follow-up seems to be of particular importance for long-term prognosis [19].

The treatment of complex cartilage lesions, especially in younger patients having expectations in his or her ability to work and quality of live represents a challenging task for the orthopaedic surgeon.

In conclusion, our results suggest that ACI is a capable of improving mid-term results in patients with complex cartilage lesions of the knee. If long term results will confirm our findings, ACI may be a valuable tool not only for focal defects but also for the treatment of complex cartilage lesions of the knee. ACI may also be used to delay the need for total knee arthroplasty.

## Competing interests

The authors declare that they have no competing interests.

## Authors' contributions

CO conceived and performed the study and drafted the manuscript. MRS participated in the design of the study, evaluation of the data and writing the manuscript. PCK, GO, AL and PPD participated in evaluation of data, drafting of the manuscript, and statistical analysis of the study. CE conceived the study, participated in its design, evaluation of data and in drafting of the manuscript. All authors read and approved the final version of the manuscript.
